# CDH6 and HAGH protein levels in plasma associate with Alzheimer’s disease in *APOE ε4* carriers

**DOI:** 10.1038/s41598-020-65038-5

**Published:** 2020-05-19

**Authors:** Shahzad Ahmad, Marta del Campo Milan, Oskar Hansson, Ayse Demirkan, Ruiz Agustin, Maria E. Sáez, Nikolaos Giagtzoglou, Alfredo Cabrera-Socorro, Margot H. M. Bakker, Alfredo Ramirez, Thomas Hankemeier, Erik Stomrud, Niklas Mattsson-Carlgren, Philip Scheltens, Wiesje M. van der Flier, M. Arfan Ikram, Anders Malarstig, Charlotte E. Teunissen, Najaf Amin, Cornelia M. van Duijn

**Affiliations:** 1 Department of Epidemiology, Erasmus Medical Center, Rotterdam, The Netherlands; 2 Neurochemistry laboratory, Department of Clinical Chemistry, Amsterdam Neuroscience, Amsterdam University Medical Centers (AUMC), Vrije Universiteit, Amsterdam, The Netherlands; 3 Clinical Memory Research Unit, Faculty of Medicine, Lund University, Lund, Sweden; 4 Memory Clinic, Skåne University Hospital, Malmö, Sweden; 5 Research Center and Memory clinic Fundació ACE. Institut Català de Neurociències Aplicades, Universitat Internacional de Catalunya, Barcelona, Spain; 6 CIBERNED, Network Center for Biomedical Research in Neurodegenerative Diseases, National Institute of Health Carlos III, Madrid, Spain; 7 Centro Andaluz de Estudios Bioinformáticos CAEBi, Sevilla, Spain; 8 Biogen Idec, Cambridge, United States; 9 Janssen Pharmaceutical NV, Turnhoutseweg 30, 2340 Beerse, Belgium; 10 Discovery Research, AbbVie Deutschland GmbH & Co. KG, Knollstrasse, 67061 Ludwigshafen, Germany; 11 Department of Neurodegeneration and Geriatric Psychiatry, University of Bonn, 53127 Bonn, Germany; 12 Division of Neurogenetics and Molecular Psychiatry, Department of Psychiatry and Psychotherapy, University of Cologne, Medical Faculty, 50937 Cologne, Germany; 13 German Center for Neurodegenerative Diseases (DZNE), 53127 Bonn, Germany; 14 Division of Systems Biomedicine and Pharmacology, Leiden Academic Centre for Drug Research, Leiden University, Leiden, The Netherlands; 15 Alzheimer center Amsterdam, Department of Neurology, Amsterdam Neuroscience, Vrije Universiteit Amsterdam, Amsterdam, UMC The Netherlands; 16 Department of Medical Epidemiology and Biostatistics, Karolinska Institutet, Stockholm, Sweden; 17 Pfizer Worldwide R&D, Stockholm, Sweden; 18 Nuffield Department of Population Health, Oxford University, Oxford, UK

**Keywords:** Alzheimer's disease, Alzheimer's disease

## Abstract

Many Alzheimer’s disease (AD) genes including Apolipoprotein E (*APOE*) are found to be expressed in blood-derived macrophages and thus may alter blood protein levels. We measured 91 neuro-proteins in plasma from 316 participants of the Rotterdam Study (incident AD = 161) using Proximity Extension Ligation assay. We studied the association of plasma proteins with AD in the overall sample and stratified by *APOE*. Findings from the Rotterdam study were replicated in 186 AD patients of the BioFINDER study. We further evaluated the correlation of these protein biomarkers with total tau (t-tau), phosphorylated tau (p-tau) and amyloid-beta (Aβ) 42 levels in cerebrospinal fluid (CSF) in the Amsterdam Dementia Cohort (N = 441). Finally, we conducted a genome-wide association study (GWAS) to identify the genetic variants determining the blood levels of AD-associated proteins. Plasma levels of the proteins, CDH6 (β = 0.638, *P* = 3.33 × 10^−4^) and HAGH (β = 0.481, *P* = 7.20 × 10^−4^), were significantly elevated in *APOE* ε4 carrier AD patients. The findings in the Rotterdam Study were replicated in the BioFINDER study for both CDH6 (β = 1.365, *P* = 3.97 × 10^−3^) and HAGH proteins (β = 0.506, *P* = 9.31 × 10^−7^) when comparing cases and controls in *APOE* ε4 carriers. In the CSF, CDH6 levels were positively correlated with t-tau and p-tau in the total sample as well as in *APOE ε4* stratum (*P* < 1 × 10^−3^). The HAGH protein was not detected in CSF. GWAS of plasma CDH6 protein levels showed significant association with a cis-regulatory locus (rs111283466, *P* = 1.92 × 10^−9^). CDH6 protein is implicated in cell adhesion and synaptogenesis while HAGH protein is related to the oxidative stress pathway. Our findings suggest that these pathways may be altered during presymptomatic AD and that CDH6 and HAGH may be new blood-based biomarkers.

## Introduction

Apolipoprotein E (*APOE*) is the most common genetic risk factor for Alzheimer’s disease (AD)^1,2^ and an important driver of the lifetime risk for AD^3,4^. *APOE* interacts with other common genetic determinants of AD^2,5^, suggesting an interaction with specific protein pathways. Despite two decades of research, the role of *APOE* in determining the risk of AD is far from being understood^6^. The IMI ADAPTED (The Alzheimer’s Disease Apolipoprotein Pathology for Treatment Elucidation and Development) is an Innovative Medicine Initiative (IMI) that aims to improve the understanding about the role of *APOE* gene in AD.

AD pathology is characterized by the extracellular deposition of amyloid-beta (Aβ)-42 and intracellular accumulation of phosphorylated tau in the brain. Cerebrospinal fluid (CSF) levels of Aβ-42, phosphorylated (p-tau) and total tau (t-tau) are well-established biomarkers of the central nervous system and brain AD pathology^7^. However, there is a growing evidence for a relation between other pathologies and AD, such as vascular pathology^8^. For example, studies integrating epidemiological and vascular research showed that vascular pathology may affect brain function and increase the risk of AD^9^. *APOE* and many of the novel genes implicated in AD are expressed in monocytes/macrophages^10–12^ in the blood, and thus these genes may alter the protein signatures in blood. There is also a growing body of evidence indicating that Aβ may disrupt the cerebral microcirculation regulation^13–15^, endothelial function^16,17^, and brain perivascular macrophages function^18^. Thus, protein and metabolite homeostasis in blood may also be altered as a consequence of (early) amyloid pathology. Indeed, there is an increasing interest in the relation between protein levels in plasma and AD during presymptomatic stages of AD^19^. Multiple studies have investigated the association of a range of proteins with AD in plasma, but few have addressed the effect of *APOE*^19–25^. Furthermore, there is lack of investigations connecting molecular signatures of AD in blood to neuropathological AD markers in CSF.

Advances in high-throughput omics technologies have allowed the detection and quantification of several classes of plasma-based biomolecular compounds including circulating metabolites and proteins^26^. In the present study, we aimed to identify altered levels of proteins in the circulation of presymptomatic AD patients in the overall population and among various genetic risk groups based on the *APOE* gene, with a view to obtaining insights into molecular signatures in the circulation. To this end, we have examined the association of neurology relevant proteins in a prospective population-based, the Rotterdam Study. Proteins associated with AD were further tested for replication in the BioFINDER study. Next, we conducted a genome-wide association study to find the genetic variants determining the blood levels of AD-associated proteins. Finally, we studied the association of the protein consistently associated with AD to amyloid and tau levels in CSF in the Amsterdam Dementia Cohort (ADC).

## Results

### Association of plasma proteins with AD

**Figure 1:**
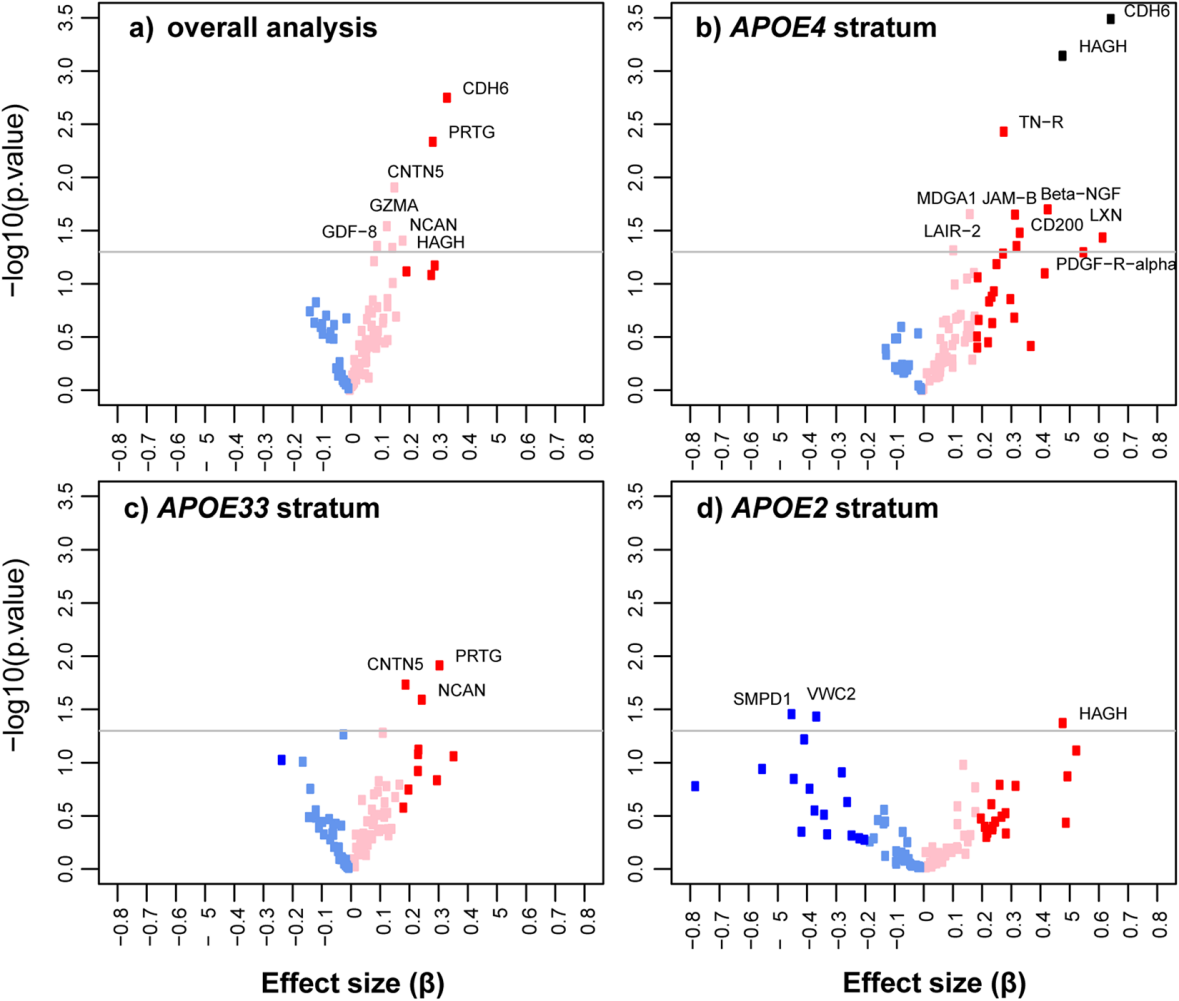
Volcano plots representing the association of plasma protein levels with Alzheimer’s disease (AD) in **(a)** overall analysis; **(b)** *APOE4* stratum; **(c)** *APOE33* stratum and **(d)** *APOE2* stratum. Each dot represents a protein with regression coefficient (β) of association plotted on x-axis and -log10 of *P-values* on y-axis. Proteins showing nominal association (*P-value* < 0.05) are annotated in overall and stratified analysis. Light blue color of dot indicates decreased protein levels with β −0.0 to −0.184 and dark blue to indicate with β < −0.184 while pink color indicates increased protein levels with β ranging from 0.0 to 0.184 and red color shows β > 0.184. Black dots are used for proteins which pass the multiple testing (false discovery rate <0.05).

Detailed results of overall and *APOE* stratified association analysis of proteins with AD are provided in Table 1 and Fig. 1. No significantly associated protein to AD was identified in the overall analysis at an *FDR* < 0.05. Overall, there is a tendency that protein levels are more likely increased (positive effect size, β) than decreased (negative effect size, β) in AD patients that carry the *APOE* ε4 allele (Fig. 1a,b) and those homozygous for *APOE* ε3 allele (Fig. 1c) but not for *APOE* ε2 patients (Fig. 1d). In *APOE* stratified discovery analysis, we observed that levels of CDH6 (β = 0.638, *P* = 3.33 × 10^−4^, *FDR* = 0.030) and HAGH (β = 0.481, *P* = 7.20 × 10^−4^, *FDR* = 0.033) were significantly increased in AD patients who carry the *APOE* ε4 allele (see Table 1). Both CDH6 (β = 0.624, *P* = 5.52 × 10^−4^, *FDR* = 0.030) and HAGH (β = 0.491, *P* = 6.62 × 10^−4^, *FDR* = 0.030) proteins remained significantly associated with AD even after adjusting for other covariates in model 2 (Supplementary Table 1). In the replication analysis in the BioFINDER study (Table 2), plasma levels of CDH6 and HAGH were significantly associated with AD in the overall sample (CDH6: β = 1.212, *P* = 5.18 × 10^−4^; HAGH: β = 0.631, *P* = 7.56 × 10^−15^) as well as in *APOE* ε4 carriers (CDH6: β = 1.365, *P* = 3.97 × 10^−3^; HAGH: β = 0.506, *P* = 9.31 × 10^−7^) but not in *APOE* ε2 carriers. Plasma levels of HAGH protein were also associated with AD in *APOE* ε33 carriers (β = 0.739, *P* = 3.76 × 10^−7^) but in this subgroup no association was seen with CDH6.

**Table 1:** Results of plasma-based proteome association with Alzheimer’s disease.

Uniprot id	Annotation	Effect size (β)	OR	SE	*P-value*	*FDR-value*
**Overall population**						
P55285	CDH6	0.334	1.397	0.106	1.78 × 10^−3^	0.162
Q2VWP7	PRTG	0.286	1.331	0.100	4.62 × 10^−3^	0.210
O94779	CNTN5	0.155	1.168	0.061	1.24 × 10^−2^	0.377
P12544	GZMA	0.129	1.138	0.059	2.88 × 10^−2^	0.598
O14594	NCAN	0.183	1.201	0.088	3.93 × 10^−2^	0.598
O14793	GDF-8	0.095	1.100	0.047	4.42 × 10^−2^	0.598
Q16775	HAGH	0.147	1.158	0.074	4.60 × 10^−2^	0.598
***APOE4*** **stratum**						
P55285	CDH6	0.638	1.893	0.171	3.33 × 10^−4^	0.030
Q16775	HAGH	0.481	1.618	0.138	7.20 × 10^−4^	0.033
Q92752	TN-R	0.280	1.323	0.094	3.72 × 10^−3^	0.113
P01138	Beta-NGF	0.431	1.539	0.182	2.00 × 10^−2^	0.340
Q8NFP4	MDGA1	0.164	1.178	0.070	2.22 × 10^−2^	0.340
P57087	JAM-B	0.318	1.374	0.137	2.24 × 10^−2^	0.340
P41217	CD200	0.335	1.398	0.155	3.31 × 10^−2^	0.394
Q9BS40	LXN	0.619	1.857	0.292	3.67 × 10^−2^	0.394
P16234	PDGF-R-alpha	0.324	1.383	0.159	4.42 × 10^−2^	0.394
Q6ISS4	LAIR-2	0.107	1.113	0.053	4.84 × 10^−2^	0.394
***APOE33*** **stratum**						
Q2VWP7	PRTG	0.309	1.362	0.122	1.22 × 10^−2^	0.778
O94779	CNTN5	0.193	1.213	0.081	1.85 × 10^−2^	0.778
O14594	NCAN	0.248	1.281	0.110	2.57 × 10^−2^	0.778
P55285	CDH6	0.202	1.224	0.150	1.79 × 10^−1^	0.842
Q16775	HAGH	−0.018	0.982	0.092	8.42 × 10^−1^	0.957
***APOE2*** **stratum**						
P17405	SMPD1	−0.447	0.640	0.203	3.51 × 10^−2^	0.966
Q2TAL6	VWC2	−0.362	0.696	0.166	3.69 × 10^−2^	0.966
Q16775	HAGH	0.482	1.619	0.227	4.25 × 10^−2^	0.966
P55285	CDH6	0.498	1.645	0.324	1.35 × 10^−1^	0.966

Abbreviations: β, regression coefficient; OR, odds ratio; SE, standard error; *APOE*, apolipoprotein E; *FDR*, False discovery rate.

Note: Multiple testing correction by false discovery rate (*FDR*) < 0.05 was considered significant.

**Table 2:** Association of plasma levels of CDH6 and HAGH proteins with Alzheimer’s disease in the BioFINDER Study.

Biomarkers*	Overall			*APOE4* stratum			*APOE33* stratum			*APOE2* stratum		
	β	SE	*P-value*	β	SE	*P-value*	β	SE	*P-value*	β	SE	*P-value*
CDH6	1.212	0.349	5.18 × 10^−4^	1.365	0.474	3.97 × 10^−3^	0.961	0.585	1.01 × 10^−1^	2.612	3.210	4.16 × 10^−1^
HAGH	0.631	0.081	7.56 × 10^−15^	0.506	0.103	9.31 × 10^−7^	0.739	0.145	3.76 × 10^−7^	5.565	3.979	1.62 × 10^−1^

Abbreviations: AD, Alzheimer’s disease; β, regression coefficient; SE, standard error.

*Logistic regression analysis adjusting for age, sex and date of sample collection.

**Figure 2:**
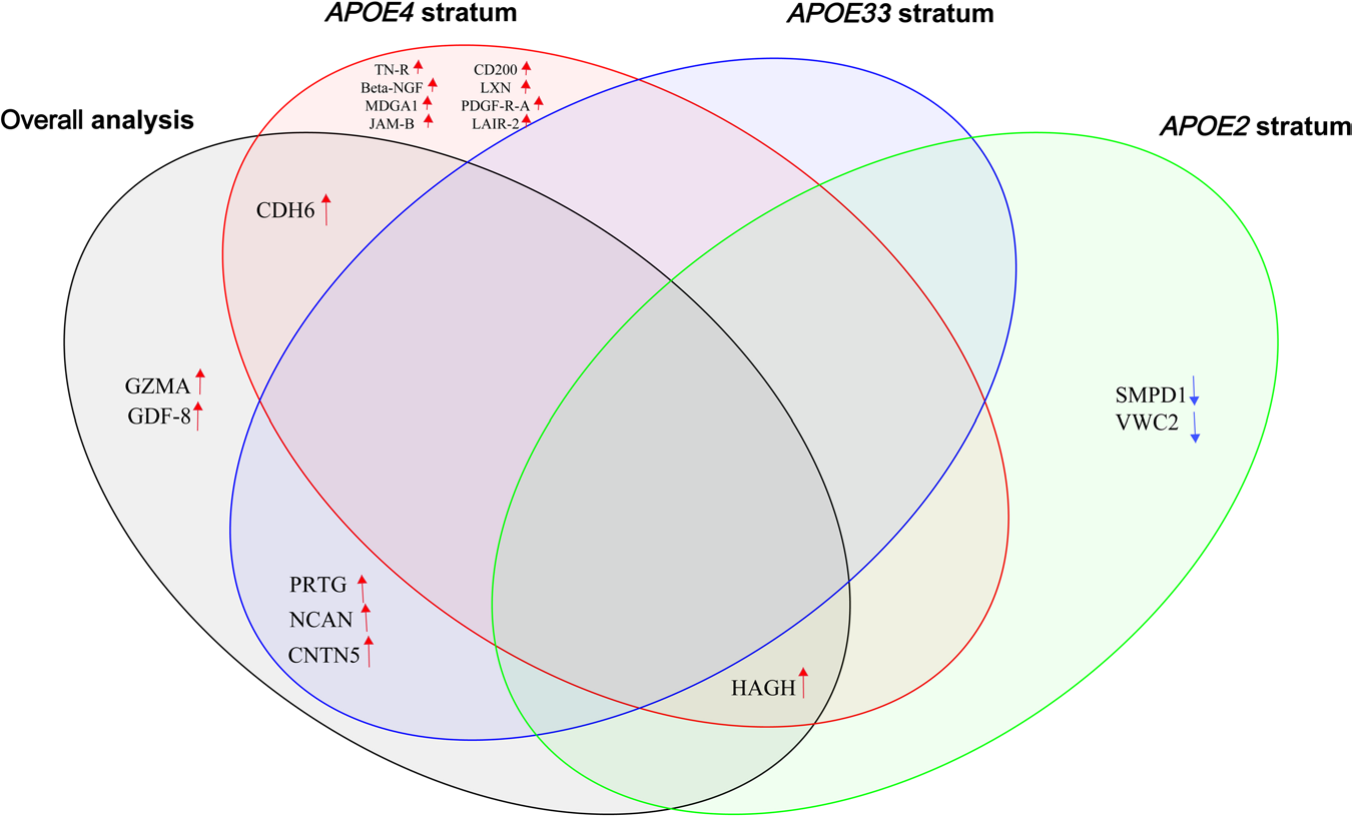
Venn diagram showing the overlap of proteins identified in the association analysis results of overall and *APOE* stratified discovery analysis.

Figure 2 shows that the *APOE* genotype modifies the association between proteins and AD based on nominal statistical significance. In discovery analysis, eight additional proteins (TN-R, Beta-NGF, MDGA1, JAM-B, CD200, LXN, PDGF-R-alpha, and LAIR-2) were also positively associated with AD *APOE* ε4 carriers (β > 0.107, *P* < 0.05), but they did not survive multiple testing. In the *APOE2* stratum, the levels of two proteins including SMPD1 and VWC2 were reduced in AD cases compared to the *APOE* genotype matched controls (Supplementary Fig. 1). In *APOE33* stratum, PRTG, CNTN5 and NCAN proteins, that do not emerge in the *APOE4* or *APOE2* stratum (see Fig. 2), showed suggestive associations but did not survive multiple testing (β > 0.193, *P* < 2.57 × 10^−2^). Both CDH6 (β = 0.202, *P* = 1.79 × 10^−1^) and HAGH (β = −0.018, *P* = 8.42 × 10^−1^) did not show association with AD in *APOE33* carriers while HAGH showed nominal association in *APOE* ε2 carriers (β = 0.482, *P* = 4.25 × 10^−2^) (See Table 1).

### Sensitivity analyses

Sensitivity analyses were performed in the Rotterdam Study to test the robustness of our findings. In the first sensitivity analysis (Supplementary Table 2), we adjusted for follow-up time, taking into account that some cases or controls may die of other diseases. This analysis showed that levels of both HAGH (β = 0.477, *P* = 6.47 × 10^−4^) and CDH6 (β = 0.661, *P* = 1.48 × 10^−4^) proteins were significantly increased in AD patients compared to controls in *APOE* ε4 carriers. In the second sensitivity analysis, we only analyzed protein that were assessed directly (non-imputed data). Similarly, the association of HAGH and CDH6 proteins with AD remained significant in *APOE* ε4 carriers when analyzing non-imputed proteomics data (Supplementary Table 3). Last but not least, we performed a formal interaction test to evaluate the interaction of *APOE* with each of the 91 proteins (Supplementary Table 4). Only three of 91 proteins showed interaction with *APOE* (*P-value* < 0.05) including HAGH (β_interaction_ = 0.414, *P*_*interaction*_ = 1.70 × 10^−2^), G-CSF (β_interaction_ = 0.276, *P*_*interaction*_ = 2.78 × 10^−2^) and CRTAM (β_interaction_ = −0.221, *P*_*interaction*_ = 3.77 × 10^−2^). Except, HAGH other two proteins (G-CSF, CRTAM) did not show association with AD in any of the analyzed *APOE* stratum (*P-value* > 0.05). For CDH6, the test for interaction was not significant (β_interaction_ = 0.078, *P*_*interaction*_ = 7.23 × 10^−1^).

### Association of CDH6 and HAGH protein levels with Aβ−42, p-tau, and t-tau in CSF

**Figure 3:**
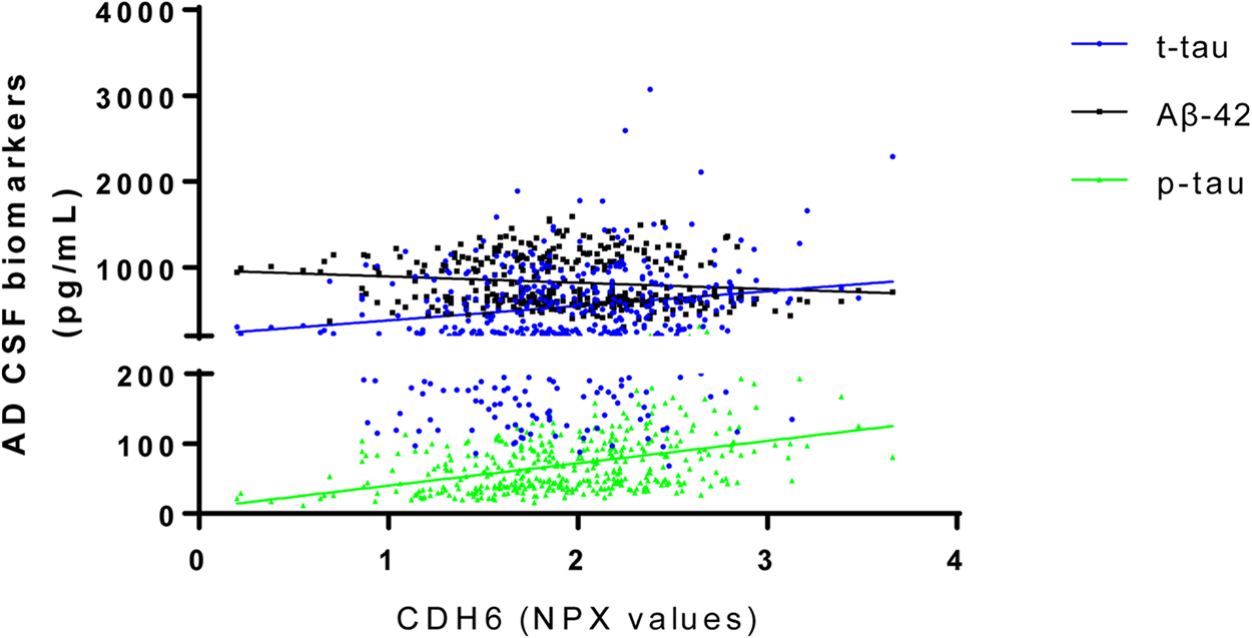
Correlation plot between cerebrospinal fluid (CSF) levels of CDH6 with Aβ−42, p-tau and t-tau.

Among the two proteins that were associated to the future risk of AD, CDH6 and HAGH, the latter was not detected in the CSF in >90% of the subjects in ADC cohort. CSF CDH6 protein levels were not associated with AD (β = 0.329, SE = 0.220, *P* = 0.136) in the overall as well as in *APOE* stratified analysis (*P* > 0.114; see Supplementary Table 5). However, multiple regression analysis adjusted for age and sex revealed a significant association of CDH6 CSF levels with both p-tau (β = 23.2, SE = 3.4, *P* = 3.48 × 10^−11^) and t-tau (β = 207.4, SE = 36.4, *P* = 2.40 × 10^−8^) when pooling AD patients and controls (Table 3 and Fig. 3). In the *APOE* stratified analysis, levels of CDH6 were significantly associated with p-tau and t-tau levels but not with Aβ−42 levels in CSF in three *APOE* strata (see Table 3). When stratifying by case-control status (Supplementary Table 6), CDH6 levels were significantly associated with p-tau and t-tau levels in both cases and controls. In controls, also Aβ−42 was positively associated with CDH6 (*P* < 1 × 10^*−3*^; see Supplementary Table 6**)**.

**Table 3:** Association of CSF based CDH6 protein levels with AD biomarkers in Amsterdam Dementia Cohort.

Biomarkers*	Overall			*APOE4* stratum			*APOE33* stratum			*APOE2* stratum		
	β	SE	*P-value*	β	SE	*P-value*	β	SE	*P-value*	β	SE	*P-value*
Aβ-42	16.574	26.827	5.37 × 10^−1^	33.701	35.001	3.37 × 10^−1^	14.499	44.966	7.48 × 10^−1^	−103.767	69.354	1.45 × 10^−1^
p-tau	23.189	3.404	3.48 × 10^−11^	26.674	5.781	8.36 × 10^−6^	21.981	5.564	1.17 × 10^−4^	25.663	7.697	2.29 × 10^−3^
T-tau	207.396	36.437	2.40 × 10^−8^	235.009	59.158	1.10 × 10^−4^	193.859	64.068	2.90 × 10^−3^	298.437	86.637	1.66 × 10^−3^

Abbreviations: AD, Alzheimer’s disease; β, regression coefficient; SE, standard error.

*Linear regression analysis adjusting for age and sex.

### Association of proteins with *APOE*

Next, we associated the *APOE* genotype to the protein levels significantly associated with AD (CDH6 and HAGH). Results of the association of protein levels with *APOE* genotypes are provided in Supplementary Table 7 and Supplementary Fig. 2. In the overall sample, CDH6 protein levels were increased in the *APOE* ε4 carriers compared to *APOE* ε2 carriers (β = 0.163, *P* = 3.79 × 10^−3^). In controls, levels of CDH6 protein were decreased (β = −0.131, *P* = 0.026) in *APOE* ε2 carriers compared to *APOE 33* carriers. In the controls, levels of HAGH were decreased in the *APOE* ε4 (β = −0.192, *P* = 0.028) and *APOE* ε2 carriers (β = −0.214, *P* = 0.042) compared to *APOE* 33 carriers.

### Genome-wide association study (GWAS)

The GWAS was conducted to determine the genetic drivers of the CDH6 and HAGH protein levels (Supplementary Figs. 3a and 4 respectively). We identified 13 genome-wide significant cis protein quantitative trait loci (pQTLs) located at 5p13.3 locus of chromosome 5 for CDH6 protein levels. All genome-wide significant pQTLs are located in the intergenic region at 5′ UTR region of the *CDH6* gene. Among the 13 identified pQTLs, rs111283466 was the lead pQTL with the effect estimate (β) of 1.068 and *P*-value 1.92 × 10^−9^ (Supplementary Table 8). Q-Q plot (Supplementary Fig. 5a) indicates that the results are well adjusted for population stratification (λ = 1.0056). Further lookups in the GTEx database showed that the lead pQTL (rs111283466) also affects the expression of *CDH6* gene in various body tissues. GWAS analysis of HAGH protein levels did not identify any genome-wide significant pQTLs. Manhattan plot and Q-Q plot for GWAS results of HAGH protein levels are provided in the Supplementary materials (Supplementary Figs. 4 and 5b).

## Discussion

In our study, plasma levels of CDH6 and HAGH proteins are significantly increased in presymptomatic AD patients compared to controls in the *APOE4* stratum. In the replication analysis, both CDH6 and HAGH proteins showed significant association with AD in the BioFINDER study in *APOE ε4* carriers. CDH6 protein levels were significantly correlated with p-tau and t-tau measurements in CSF of the ADC. In GWAS analysis, we have also identified a genome-wide significant pQTL for CDH6 protein levels in the blood (rs111283466), which also affects the expression levels of *CDH6* transcripts in several tissues.

We observed a significant increase of CDH6 protein levels in the plasma of presymptomatic AD cases carrying the *APOE* ε4 allele which was also replicated in the BioFINDER study. When comparing our findings to the other studies^19–22^, we do not have an overlap in understudy proteins. However, like previous studies we do find an effect of the *APOE* gene on plasma level of prorteins^21,22^. In the *APOE*4 stratum, we see that the volcano plot (Fig. 1) is clearly asymmetric suggesting increased levels of most neuronal proteins in AD patients carrying this allele before the clinical onset of disease. This might be explained by an increase in the blood-brain barrier permeability in *APOE* ε4 carriers^27^, which may lead to increased levels of CDH6 in the blood as a result of higher levels of CDH6 in the brain. We found that CDH6 levels in the blood are driven by a genetic variant (rs111283466) in the cis-regulatory region. This may determine the CDH6 levels in both brain and blood cells, leaving the possibility open that elevated CDH6 has a blood-derived origin. Yet, such a mechanism does not explain why elevated levels in the blood are only seen in patients carrying the *APOE* ε4 allele. It is of note that the CDH6 coding gene is part of a larger cluster of cadherin (CDH) genes including *CDH9, CDH*10*, CDH12 and CDH18*. As all of the CDH genes are paralogues and share homology, it is crucial to exclude cross-reactions of the antibodies^28^ used by Olink across CDH proteins. Our GWAS benchmarks that the protein assessed in our plasma is indeed CDH6, as we found that the most important driver of the protein is in the promoter region of *CDH6*. None of the recently published GWAS of proteins reported significant pQTLs for CDH6 protein based on SomaLogic^29,30^. The aptomere based measurement of the SomaLogic yields a different protein spectrum than that of the antibody-based method of Olink^31^. Our identified pQTLs did not show any association with AD (*P-value* < 0.05) in the largest AD GWAS^11^. However, we find in our study that levels of CDH6 were increased in *APOE* ε4 carriers compared to *APOE* ε2 carriers. We do find that the region is associated to postcentral gyri in GWAS^32^ and a study has reported reduced volume of postcentral gyri in dementia patients^33^.

Interestingly, we found significant positive associations of CSF levels of CDH6 protein with p-tau and t-tau levels in overall as well as in AD and controls only analyses, which are considered as biomarkers of neuronal injury and tau pathology^34,35^. The upregulation of CDH6 protein in cerebral cortices of AD mice models (*APP/PS1*) compared to wild type has been reported by Lu *et al*.^36^, which is in line with our observation of positive correlation between CDH6 levels and AD pathological markers in CSF. Yet, we did not observe an association of the CDH6 protein with AD in CSF. We also observed positive association between CDH6 levels with amyloid-beta 42 in CSF of the controls, which might indicate disturbance in the amyloid-beta 42 metabolism which precedes decades before the buildup of Aβ in the brain^37^. Alternatively, it may point towards a similar mechanism of production of the Aβ-42 and CDH6 proteins in a healthy state^38^. Increased levels of phosphorylated CDH6 protein levels were reported upon the addition of amyloid-beta in cortical neuronal cells^39^, which adds evidence to the role of CDH6 in AD pathology. Taken together, these findings suggest that CSF levels of CDH6 protein may be associated with neuronal and axonal cell injury and neurofibrillary tangles in AD.

CDH6 is a cell surface glycoprotein that belongs to type II cadherin’s^40^. Cadherins are highly expressed in the brain and other tissues. They strongly interact with other molecules to perform molecular processes including synaptic functions^41–43^, synaptogenesis^44^, TGF-B signaling^45^, neural crest differentiation^46^, presenilin-mediated signaling and integrity of blood-brain barrier^47^. Although it is not possible to infer whether the correlation of AD pathology with CDH6 in plasma and CSF, are cause or consequence of the disease, several pieces of evidence favor the role of CDH6 in the pathogenesis of AD^39,48^. A recent study showed that the ADAM10 enzyme, whose coding gene is associated with AD^11^, is involved in proteolytic cleavage of the *CDH6* protein, resulting in the formation of C-terminal fragment^49^, in a similar manner as it cleaves the amyloid precursor protein (*APP*)^50,51^. The transmembrane N-cadherin (*CDH2*), a paralogue of CDH6 and functionally related to CDH6^43^, is also known to be cleaved by ADAM10 into N-cadherin C-terminal fragment 1 (NcadCTF1). Andreyeva *et al*.^52^, have demonstrated that NcadCTF1 leads to accelerated amyloid-β-induced synaptic impairment, a process that characterizes an early stage event in AD^53,54^. Increased levels of NcadCTF1 were also found in postmortem AD brain tissues compared with controls, suggesting that cadherins might induce synaptic dysfunction in a synergistic manner^52^.

In addition to CDH6, increased plasma levels of HAGH (Hydroxyacylglutathione hydrolase, mitochondrial) protein also showed significant association with AD in those who carry the *APOE* ε4 variant and suggestive association in overall and in *APOE* ε2 carriers. This finding is in line with the recently published findings of the BioFINDER study^25^ and further supported by the *APOE* stratified analysis in the BioFINDER study that was conducted for the present study. In the replication analysis, plasma levels of HAGH showed significant association in both *APOE* ε4 and *APOE* ε33 carriers while in the discovery analysis in the Rotterdam Study HAGH only showed significant association in *APOE* ε4 carriers which may be due to the lack of power. The HAGH protein is also known as glyoxalase-2, an enzyme, which is involved in the glyoxalase system along with glyoxalase-1 and its cofactor glutathione, a key player is oxidative stress control^55,56^. Overall, the glyoxalase system is involved in the detoxification of glycolysis by-products particularly cytotoxic metabolite methylglyoxal^57^. Levels of methylglyoxal in plasma are elevated during various disease conditions including hyperglycemia, which leads to the formation of reactive oxygen species (ROS) and causes oxidative stress. Moreover, methylglyoxal is also the precursor of glycation end products (AGEs) which are implicated in neurodegeneration and AD^58,59^. The most compelling evidence for the role of the glyoxalase-2 protein in AD is that the AGEs and glyoxalase system is implicated in the regulation of amyloid precursor protein (*APP*) expression^60,61^. Although glyoxalase system attributes protection against methylglyoxal mediated oxidative stress, earlier studies have also observed increased levels of glyoxalase-1 enzyme (involved in the first step of methylglyoxal detoxification) in early AD stages^62,63^. Increased levels of glyoxalase-2 (involved in the second step of methylglyoxal detoxification) in plasma might be a compensatory mechanism to increased levels of methylglyoxal during the early phase of disease or a general stress response^55^. The growing number of studies have suggested the involvement of oxidative stress during the prodromal stage of AD^64–66^, which is in line with our finding of increased levels of glyoxalase-2 observed before the onset of AD.

The strength of the current study includes that it is conducted in the prospective population-based RS cohort, where samples were selected with mean 6.9 years of follow-up preceding the diagnosis of AD. It allowed us to study the plasma proteomics changes prior to the development of AD clinical symptoms. As AD is a disorder of the brain, we have validated that CSF levels of CDH6 are also associated with biomarkers of AD in CSF in an independent cohort. Further, we used the Olink neurology proteomic panel of 91 proteins for the quantification of proteins in the plasma, which estimates targeted proteins expressed in the brain from different pathways. One of the major limitations of our study is the limited sample size, including a small number of *APOE ε4* carrier controls in the stratified analysis.

In conclusion, we observed elevated protein levels of CDH6 in plasma of AD patients carrying *APOE ε4* allele in the discovery and replication analysis, a protein that plays a role in synaptogenesis. Positive correlation of CSF CDH6 levels with p-tau and t-tau may also indicate the association of CDH6 with neurodegeneration. We further found the association of the plasma levels of HAGH protein to AD in those carrying the *APOE* ε4 allele. Association of HAGH with AD further suggest the involvement of the glyoxalase and oxidative stress pathways in the pathogenesis of AD.

## Methods

### Study populations

#### Rotterdam study

The Rotterdam Study (RS) is a prospective population-based study comprising of 14,926 middle and older aged (≥45 years) individuals from the Ommoord district of Rotterdam. The RS consists of three cohorts including RS-I (started in 1990, N = 7983 participants), RS-II (started in 2000, N = 3011) and RS-III (started in 2006, N = 3932)^67^. Study participants were extensively interviewed and physically examined at baseline and after every 3 to 4 years. For each participant fasting blood was collected at a dedicated center, centrifuged (Speed = 3500 g for 20 min at 4 °C) within 4 hours of venipuncture to collect plasma and stored at −80 °C. The study has been approved by the Medical Ethical Committee of Erasmus Medical Center and by the Ministry of Health, Welfare and Sport of the Netherlands. Written informed consent was obtained from each study participant to participate and to collect information from their treating physicians. All methods were performed in accordance with the relevant guidelines and regulations. In current nested case-control proteomics analysis, we chose 161 incident AD cases and 155 controls match with respect to their age and sex, from the fifth visit of RS-I (RS-I-5) cohort. Table 4 shows the baseline characteristics of the selected sample. There were no significant differences in age, sex and body mass index (BMI). AD patients were more often carriers of the *APOE* ε4 variant and less often of the *APOE* ε2 variant. Blood for the proteome profiling was collected on average 6.9 years (standard deviation [SD] = 1.7) before the onset of clinical dementia in patients and mean 8.7 years (SD = 3.2) before the latest follow-up in controls.

**Table 4:** Population descriptive of the Rotterdam Study.

N	Total participants	Incident AD cases	Controls
	316	161	155
Age (SD) blood collection, years	77.16 (5.39)	77.43 (5.21)	76.89 (5.59)
Age at onset/last follow-up (SD)	84.99 (5.33)	84.37 (5.01)	85.63 (5.56)
Female (%)	201 (63%)	104 (65%)	97 (63%)
Body Mass index (SD)	27.32 (4.10)	27.29 (3.75)	27.37 (4.46)
Follow-up (SD) years	7.82 (2.71)	6.94 (1.71)	8.74 (3.22)
***APOE*** **genotype**			
*APOE* 44/34/24	98	68	30
*APOE* 33	171	76	95
*APOE* 22/23	34	13	21

Abbreviations: AD, Alzheimer’s disease, SD, Standard deviation, *APOE*, apolipoprotein E gene.

#### Dementia diagnosis

Over time, all participants were screened for dementia using the Mini-Mental State Examination (MMSE)^68^ and Geriatric Mental Schedule (GMS)^69^ organic level for all participants. Screen-positive subjects (MMSE < 26 or GMS organic level > 0) underwent the Cambridge examination for mental disorders of the elderly (CAMDEX)^70^ and participants suspected of having dementia were extensively examined with neuropsychological testing and neuroimaging biomarkers when available. Patients were further ascertained by linking them with their medical records from general practitioners, the regional institute for outpatient mental health care and municipality. Dementia of all patients was diagnosed based on the internationally accepted Diagnostic and Statistical Manual of Mental Disorders (DSM-III-R) criteria and AD using the National Institute of Neurological Disorders and Stroke–Association Internationale pour la Recherche et l’Enseignement en Neurosciences (NINCDS-ADRDA)^71^ criteria for possible, probable and definite AD. NINCDS-ADRDA criteria were also used to diagnose vascular dementia. The final diagnosis was confirmed by a panel of neurologists, neurophysiologists, and research physicians^72^. AD diagnosis in RS is also provided in more detail in earlier publications^72^.

#### Proteome profiling

Proteomics profiling of the 316 plasma samples was performed using neurology panel of OLINK’s Proximity Extension Assay (ProSeek, OLINK AB, Uppsala, Sweden), which includes 91 proteins involved in various pathways including axon development, axon guidance, cell adhesion, cell death, cell differentiation, cell growth, cellular metabolic process, immune response, MAPK cascade, neurogenesis, proteolysis, signal transduction and synapse assembly (https://www.olink.com/products/neurology/). This method uses affinity-based assay, in which a pair of oligonucleotide-labeled antibody probes bind to a target protein. Proximity-dependent DNA polymerization event forms a polymerase chain reaction (PCR) target sequence between two probes bound in close proximity. The generated PCR target sequence is detected and quantified using real-time PCR method. The resultant protein abundance is provided as NPX (Normalized Protein Expression), which is an arbitrary unit on log2 scale. Lower limit of detection is estimated based on negative controls inserted in each run and measurements below this limited were treated as missing. None of the detected markers in our dataset reach missingness more than 10 percent. Protein markers with missing values less than 10% were imputed with the lowest detected limit for further analysis. More detailed information about detection limits, assay performance and validation methods are available from the service provider (www.olink.com)^73^.

#### *APOE* genotyping

In the RS *APOE* genotyping was performed using Polymerase chain reaction (PCR) and amplified PCR product was digested with *HhaI* enzyme. Restriction fragments of enzyme products were visualized by silver staining after getting them separated with precast ExcelGel gels (Pharmacia Biotech, Uppsala, Sweden). Genotype results were examined by three independent persons. In the case of non-agreement *APOE* genotype was repeated^74,75^.

#### Genotyping and imputations

In the RS participant’s blood was collected during baseline and follow-up visit. DNA genotyping was performed for all the participants with proper DNA quality with the 550 K, 550 K duo, or 610 K Illumina arrays. In genotyping quality control, genetic variants exclusion criteria include, call rate <95%, Hardy-Weinberg equilibrium *P* < 1.0 × 10^−6^ and Minor Allele Frequency (MAF) < 1%. Sample exclusion criteria include excess autosomal heterozygosity (0.336), call rate <97.5%, duplicate or family relationships and ethnic outliers identified by the identity-by-state clustering analysis (having identity-by-state probability <97% or>3 standard deviation from population mean)^76^. Further, genetic variants were imputed with the Haplotype Reference Consortium (HRC) reference panel (version 1.0)^77^, using the Michigan imputation server^78^. The server uses SHAPEIT2 (v2.r790)^79^ to phase the genotype data and performs imputation with Minimac 3 software^80^. Genotyping information was available for 281 among 316 participants included in the current study.

### BioFINDER study

In the current study, replication analysis was performed in 671 participants (AD patients = 186, Controls = 485) of the BioFINDER (Biomarkers For Identifying Neurodegenerative Disorders Early and Reliably) study. Characteristics of the BioFINDER study participants included in the replication analysis are provided in Supplementary Table 9. The BioFINDER study includes participants from southern Sweden recruited between 2009 and 2014 (www.biofinder.se). The study participants were assessed by experienced physicians including the neurological, psychiatric and cognitive assessments^81^. The NINCDS-ADRDA criteria were used to classify Alzheimer’s disease dementia patients for probable Alzheimer’s disease patients. All dementia due to Alzheimer’s disease patients had pathological CSF Aβ42/Aβ40 ratio of <0.1. The inclusion criteria for the cognitively normal elderly participants included (i) aged 60–80 years, (ii) MMSE scores ranging between 28–30 at their baseline screening visit, (iii) no cognitive impairment symptoms assessed by a physician, and (iv) not fulfilling the criteria for mild cognitive impairment or dementia. Exclusion criteria included (i) refused lumbar puncture, (ii) significant neurological or psychiatric disease, (iii) current alcohol or substance misuse, or (iv) systematic illness preventing them from participating in the study^25,81^. Written Informed consents were collected from each study participant and the study has been approved by the Regional Ethics Committee in Lund, Sweden.

#### Protein profiling

During the baseline visit of the BioFINDER study, plasma and lumbar CSF samples were collected from non-fasting participants. Standardized protocol was followed to analyze the plasma and CSF samples. All samples were centrifuged at 2000 g (+4 °C for 10 min), and aliquoted into 1 ml polypropylene tubes (Sarstedt AG & Co., Nümbrecht, Germany), and stored at −80 °C. Before the proteomics profiling, plasma and CSF samples underwent one cycle of freeze-thaw, and further aliquoted into 200 L Lobind tubes (Eppendorf Nordic A/S, Denmark). Protein concentrations were quantified using the ProSeek multiplex immunoassay, developed by Olink Proteomics (Uppsala, Sweden)^25^.

### Amsterdam dementia cohort (ADC)

In the validation analysis of most interesting proteins, we used 441 participants from the ADC cohort whose CSF samples were already profiled for neurology related proteins using the OLINK’s Proximity Extension Assay (ProSeek, OLINK AB, Uppsala, Sweden). Information about characteristics of patients included in current analysis as a part of the validation dataset is listed in Supplementary Table 10.

The ADC is a prospective memory-clinic cohort that was established in September 2000 at the Alzheimer Center Amsterdam of Amsterdam UMC. The cohort has included 6000 individuals until September 2017^82,83^. All participants underwent standardized cognitive screening including neurological and cognitive examination, blood sampling, a lumbar puncture to collect CSF and brain magnetic resonance imaging. All CSF samples were stored in agreement with the JPND-BIOMARKAPD guidelines^84^. All subjects provided written informed consent for use of biomaterial and clinical data for research and the study was approved by the local medical ethical review board. All methods were performed in accordance with the relevant guidelines and regulations. A sample of 441 participants selected for our validation analysis consists of 242 AD and 199 cognitively normal controls who were presented at the memory clinic with subjective cognitive decline (i.e., Criteria for mild cognitive impairment and dementia not fulfilled)). As additional inclusion criteria, controls were required to have normal AD CSF biomarkers profile: low CSF β-amyloid 1–42 (Aβ42) and high p- or t-tau level (applying local laboratory cut-offs) and to remain cognitively stable for 2 years. All participants underwent standard neurological and cognitive assessments and the diagnosis was assigned according to consensus AD criteria^83^. Global Mini-Mental State Examination (MMSE) was used to examine global cognition. The levels of CSF AD-related biomarkers (Aβ42, total and phosphorylated tau [t-Tau and p-Tau_181_]) were analyzed at Amsterdam UMC as part of the routine diagnostic procedure using commercially available kits (Innotest Aβ(1-42), total Tau, phospho-Tau(181 P); Fujirebio, Ghent, Belgium)^10,24^.

### Statistical analysis

#### Plasma protein association with AD

To identify AD-associated proteins, plasma levels of 91 proteins were compared between incident AD cases and non-demented controls using logistic regression, adjusted for age and sex in the first model. In the second model, we additionally adjusted for body mass index (BMI), smoking, educational status and medication use (lipid-lowering medications, antihypertensive and anti-inflammatory medication). To identify *APOE* specific associations of proteins with AD, we performed stratified association analysis based on *APOE* genotype carrier status. All participants were divided into *APOE4* stratum (*APOE* 44/34/24), *APOE3* stratum (*APOE* 33) and *APOE2* stratum (*APOE* 22/23). Participants with *APOE 24* genotypes were pooled within the *APOE4* stratum because an earlier study has demonstrated that the risk profiles of *APOE 24* genotype to AD and dementia is similar to those with *APOE 34* genotype carriers^5^. The association results were corrected for multiple testing using false discovery rate (*FDR*) by Benjamini and Hochberg method separately for the overall analysis, and in each *APOE* stratum^85^ and association tests with *FDR* < 0.05 were considered significant. All analyses were performed using R software (https://www.r-project.org).

#### Sensitivity analyses

Moreover, we performed sensitivity analyses. In the first sensitivity analysis, we repeated the overall and *APOE* stratified regression analysis (Model 1: age and sex) additionally adjusting for the follow-up time (the time between blood collection and onset of AD or last follow-up for controls). In the second sensitivity analysis, to assess the differential bias due to missingness, we performed the overall and *APOE* stratified association analysis in the non-imputed proteomics data adjusting for age and sex. We also tested the interaction of *APOE* genotype (ε4 carriers and non-carriers) and proteins levels using logistic regression model adjusting for age and sex.

#### Additional analysis of proteins showing association with AD

**Figure 4:**
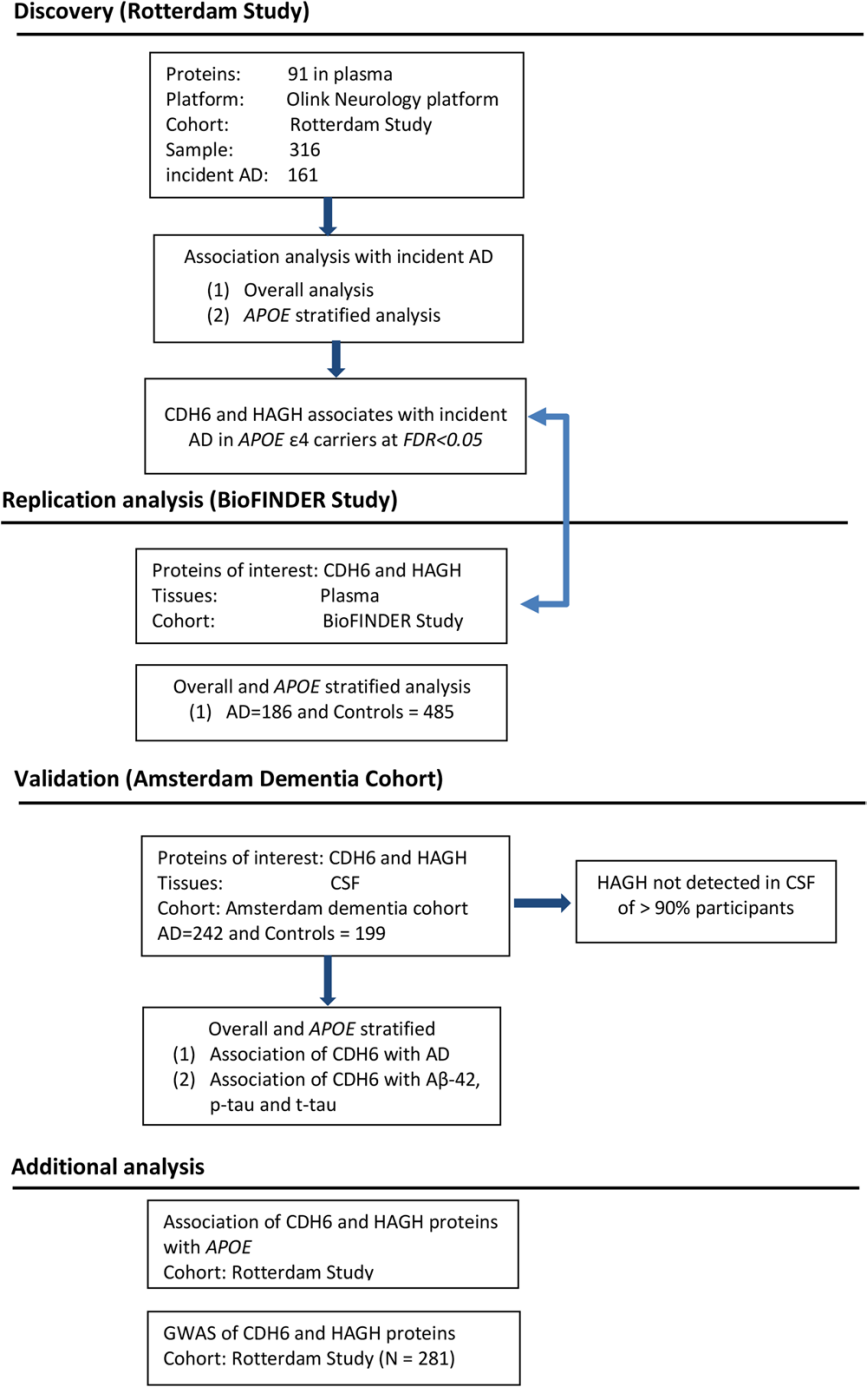
Flowchart of the analyses. Rotterdam Study was used as discovery cohort in plasma-based proteomics analysis. Altered proteins in plasma analysis were replicated in the BioFINDER study and further validated in Amsterdam Dementia Cohort participants. Abbreviations; AD: Alzheimer’s disease; *APOE*: apolipoprotein E; GWAS: genome-wide association study; CSF: cerebrospinal fluid.

A detailed flowchart of the analysis is provided in Fig. 4 about the discovery, replication and validation analysis. Proteins that appeared significantly altered in overall or *APOE* stratified analysis were further tested for association with *APOE* genotypes; second GWAS was performed to identify pQTLs, regulating the levels of protein in blood.

#### Replication analysis

Replication analysis of two proteins was performed in an independent BioFINDER study. We performed association of plasma levels of proteins with AD versus controls (AD cases = 186, controls = 485) in the overall sample and stratified by *APOE* genotype: *APOE4* stratum (*APOE* 44/34), *APOE33* stratum (*APOE* 33) and *APOE2* stratum (*APOE* 22/23). We used logistic regression analysis adjusted for age, sex and date of sample collection.

#### Validation analysis: Association of CSF protein levels with Aβ-42, p-tau, and t-tau

In the validation analysis of specific proteins in an independent ADC cohort (N = 441), we performed association of CSF protein levels with AD versus control group and with Aβ-42, p-tau and t-tau levels in CSF. All the validation analyses were performed in the overall sample and stratified by *APOE* genotype: *APOE4* stratum (*APOE* 44/34), *APOE33* stratum (*APOE* 33) and *APOE2* stratum (*APOE* 22/23). We used linear regression analysis adjusted for age and sex to evaluate the association of proteins measured in CSF with AD brain pathology biomarkers in the overall sample and stratified by clinical diagnosis (AD and controls).

#### Association of plasma protein levels with APOE genotype

To further evaluate the association of proteins with *APOE* genotypes, we compared protein levels, among *APOE* genotype groups (*APOE* 44/34/24 = *1* versus *APOE* 33 = *0*, *APOE* 44/34 = 1 versus *APOE* 22/23 = 0 and *APOE* 22/23 = 1 versus *APOE* 33 = 0) in the overall study sample, in AD patients, and in control groups separately. Linear regression analysis was performed using protein levels as outcome and *APOE* status as predictor, adjusted for age and sex.

#### Genome-wide association study

Further, we performed the genome-wide association study (GWAS) to identify protein quantitative trait loci (pQTLs) for candidate proteins. We regressed out protein levels against age, sex and principal components to calculate residuals. To normalize the calculated residuals we applied Rank-inverse transformation on residuals. Principal components derived from genotypes were used in the association analysis to adjust for population stratification. GWAS of rank-inverse normalized residuals was performed using score test option in RVTEST software^86^. Variants with low imputation quality R-squared <0.3 and minor allele count less than five were excluded from the results. Manhattan and quantile-quantile (Q-Q) plots for GWAS results were generated with web-based utility Functional mapping and annotation of genetic associations (FUMA)^87^ and regional association plots using LocusZoom (http://locuszoom.org). pQTLs with a *P*-value < 2.5  × 10^−8^ (5 × 10^−8^/2 tested proteins) were considered genome-wide significant. To check the overlap of identified pQTL with expression quantitative loci (eQTLs) we used GTEx data base^88^.

## Supplementary information

Supplementary information.

## Data Availability

Current study used data from RS and ADC, where sharing of participants data is not allowed publicly due to legal and ethical permissions. Informed consents collected for both studies do not allow to share individual participants data in public repository. Data access can be made available for interested researchers upon request to corresponding author Cornelia M. van Duijn (Cornelia.vanDuijn@ndph.ox.ac.uk).
