# Supplementary material for: CDH6 and HAGH protein levels in plasma associate with Alzheimer’s disease in APOE ε4 carriers
Source: Sci Rep. 2020 May 19;10:8233. doi: 10.1038/s41598-020-65038-5 (PMC7237496; doi:10.1038/s41598-020-65038-5)
Supplement: Supplementary file 1 — Supplementary information. [file 41598_2020_65038_MOESM1_ESM.pdf]

Supplementary Materials

CDH6 and HAGH protein levels in plasma associate with Alzheimer's disease in *APOE ε4* carriers

Authors:

Shahzad Ahmad, MSc*[1], Marta del Campo Milan, PhD[2], Oskar Hansson PhD[3,4], Ayse Demirkan, PhD[1], Ruiz Agustin, MD, PhD[5,6], Maria E Sáez, PhD[7], Nikolaos Giagtzoglou, PhD[8], Alfredo Cabrera-Socorro, PhD[9], Margot H.M. Bakker, PhD[10], Alfredo Ramirez MD, PhD[11,12,13], Thomas Hankemeier, PhD[14], Erik Stomrud, MD, PhD[3,4], Niklas Mattsson-Carlgren MD, PhD[3,4], Philip Scheltens, MD, PhD[15], Wiesje M. van der Flier, PhD[15], M. Arfan Ikram, MD, PhD[1], Anders Malarstig PhD[16,17], Charlotte E Teunissen, PhD[2], Najaf Amin, PhD[1], Cornelia M. van Duijn, PhD*[1,18]

Affiliations:

[1] Department of Epidemiology, Erasmus Medical Center, Rotterdam, The Netherlands

[2] Neurochemistry laboratory, Department of Clinical Chemistry, Amsterdam Neuroscience, Amsterdam University Medical Centers (AUMC), Vrije Universiteit, the Netherlands.

[3] Clinical Memory Research Unit, Faculty of Medicine, Lund University, Lund, Sweden

[4] Memory Clinic, Skåne University Hospital, Malmö, Sweden

[5] Research Center and Memory clinic Fundació ACE. Institut Català de Neurociències

Aplicades. Universitat Internacional de Catalunya. Barcelona. Spain.

[6] CIBERNED, Network Center for Biomedical Research in Neurodegenerative

Diseases, National Institute of Health Carlos III, Spain.

[7] Centro Andaluz de Estudios Bioinformáticos CAEBi Sevilla Spain

[8] Biogen Idec

[9] Janssen Pharmaceutical NV, Turnhoutseweg 30, 2340 Beerse, Belgium.

[10] Discovery Research, AbbVie Deutschland GmbH & Co. KG, Knollstrasse, 67061 Ludwigshafen, Germany

[11] Department of Neurodegeneration and Geriatric Psychiatry, University of Bonn, 53127 Bonn, Germany.

[12] Division of Neurogenetics and Molecular Psychiatry, Department of Psychiatry and Psychotherapy, University of Cologne, Medical Faculty, 50937 Cologne, Germany.

[13] German Center for Neurodegenerative Diseases (DZNE), 53127 Bonn, Germany.

[14] Division of Systems Biomedicine and Pharmacology, Leiden Academic Centre for Drug Research, Leiden University, Leiden, The Netherlands.

[15] Alzheimer center Amsterdam, Department of Neurology, Amsterdam Neuroscience, Vrije Universiteit Amsterdam, Amsterdam, UMC, the Netherlands

[16] Department of Medical Epidemiology and Biostatistics, Karolinska Institutet, Stockholm, Sweden.

[17] Pfizer Worldwide R&D, Stockholm, Sweden.

[18] Nuffield Department of Population Health, Oxford University, Oxford, UK

Supplementary Table 1 Results of plasma-based proteome association with Alzheimer's disease in Rotterdam Study Model 2*

Annotation	Uniport ID	Effect size (β)	OR	SE	*P-value*	*FDR-value*
**Overall population**						
CDH6	P55285	0.325	1.384	0.109	3.16E-03	0.288
PRTG	Q2VWP7	0.266	1.304	0.106	1.31E-02	0.471
GZMA	P12544	0.141	1.152	0.060	1.95E-02	0.471
CNTN5	O94779	0.148	1.160	0.064	2.07E-02	0.471
***APOE4* stratum**						
CDH6	P55285	0.624	1.867	0.174	5.52E-04	0.030
HAGH	Q16775	0.491	1.633	0.139	6.62E-04	0.030
Beta-NGF	P01138	0.491	1.633	0.184	9.27E-03	0.184
JAM-B	P57087	0.365	1.441	0.139	1.03E-02	0.184
MDGA1	Q8NFP4	0.186	1.204	0.073	1.26E-02	0.184
PLXNB3	Q9ULL4	0.416	1.515	0.166	1.39E-02	0.184
CDH3	P22223	0.347	1.415	0.138	1.42E-02	0.184
TN-R	Q92752	0.241	1.272	0.100	1.79E-02	0.203
PDGF-R-alpha	P16234	0.376	1.456	0.167	2.69E-02	0.272
LAIR-2	Q6ISS4	0.112	1.119	0.054	4.01E-02	0.365
LXN	Q9BS40	0.622	1.863	0.307	4.55E-02	0.377
***APOE33* stratum**						
PRTG	Q2VWP7	0.326	1.386	0.136	1.74E-02	0.884
CNTN5	O94779	0.199	1.220	0.086	2.26E-02	0.884
***APOE2* stratum**						
SMPD1	P17405	-0.491	0.612	0.206	2.52E-02	0.775
RGMA	Q96B86	0.609	1.839	0.285	4.30E-02	0.775
CLM-1	Q8TDQ1	0.303	1.354	0.143	4.44E-02	0.775
SCARF2	Q96GP6	0.623	1.864	0.300	4.89E-02	0.775

Abbreviations: β, regression coefficient; OR, odds ratio; SE, standard error; *APOE*, apolipoprotein E; FDR, False discovery rate
*Model 2 was adjusted for age, sex, BMI, smoking (current versus non-smokers), educational attainment (high, medium, low) and medication intake (lipid lowering, anti-hypertensive, and anti-inflammatory), N = 300 (AD = 156, Control = 144)

Supplementary Table 2: Results of plasma-based proteome association with Alzheimer's disease in Rotterdam Study adjusting for age, sex and followup time

Uniprot id	Annotation	Effect size (β)	SE	*P-value*	*FDR-value*
**Overall population**					
P55285	CDH6	0.331	0.100	1.05E-03	5.72E-02
O14793	GDF-8	0.145	0.045	1.26E-03	5.72E-02
O94779	CNTN5	0.143	0.058	1.45E-02	3.78E-01
Q14108	SCARB2	-0.180	0.075	1.66E-02	3.78E-01
Q9BS40	LXN	0.327	0.152	3.18E-02	3.86E-01
Q9BZZ2	SIGLEC1	-0.124	0.058	3.50E-02	3.86E-01
Q16775	HAGH	0.141	0.069	4.30E-02	3.86E-01
P21757	MSR1	-0.150	0.074	4.38E-02	3.86E-01
Q2MKA7	RSPO1	-0.166	0.083	4.63E-02	3.86E-01
Q8NFP4	MDGA1	0.085	0.042	4.64E-02	3.86E-01
Q2VWP7	PRTG	0.193	0.097	4.66E-02	3.86E-01
***APOE*4 stratum**					
P55285	CDH6	0.661	0.167	1.48E-04	1.35E-02
Q16775	HAGH	0.477	0.135	6.47E-04	2.95E-02
Q92752	TN-R	0.256	0.094	7.85E-03	2.38E-01
Q9BS40	LXN	0.746	0.288	1.11E-02	2.52E-01
Q8NFP4	MDGA1	0.165	0.069	1.87E-02	3.40E-01
P41217	CD200	0.349	0.152	2.35E-02	3.57E-01
Q16620	NTRK2	0.608	0.274	2.89E-02	3.69E-01
P57087	JAM-B	0.291	0.136	3.52E-02	3.69E-01
P14384	CPM	0.496	0.234	3.65E-02	3.69E-01
Q96B86	RGMA	0.287	0.138	4.08E-02	3.72E-01
P09919	G-CSF	0.198	0.098	4.72E-02	3.76E-01
***APOE33* stratum**					
Q14108	SCARB2	-0.241	0.086	5.63E-03	3.15E-01
O14594	NCAN	0.270	0.099	6.92E-03	3.15E-01
O94779	CNTN5	0.166	0.073	2.41E-02	4.14E-01
Q9HAN9	NMNAT1	0.113	0.053	3.40E-02	4.14E-01
O14793	GDF-8	0.134	0.064	3.71E-02	4.14E-01
Q92752	TN-R	0.139	0.066	3.76E-02	4.14E-01
Q96GW7	BCAN	0.171	0.082	3.86E-02	4.14E-01
P37023	SKR3	-0.219	0.110	4.79E-02	4.14E-01
Q9NP84	TNFRSF12A	-0.190	0.096	4.97E-02	4.14E-01
APOE2 stratum					
Q92765	sFRP-3	-0.544	0.218	1.87E-02	9.88E-01
P17405	SMPD1	-0.453	0.204	3.41E-02	9.88E-01
Q16775	HAGH	0.504	0.229	3.58E-02	9.88E-01
Q2TAL6	VWC2	-0.352	0.171	4.83E-02	9.88E-01

Abbreviations: β, regression coefficient; SE, standard error; *APOE*, apolipoprotein E; FDR, False discovery rate
*adjusted for age, sex, follow-up time

Supplementary Table 3: Results of plasma-based proteome association with Alzheimer's disease in Rotterdam Study without imputations

Uniprot id	Annotation	Effect size (β)	SE	*P-value*	*FDR-value*
**Overall population**					
P55285	CDH6	0.334	0.106	1.78E-03	1.60E-01
Q2VWP7	PRTG	0.286	0.100	4.62E-03	2.08E-01
O94779	CNTN5	0.155	0.061	1.24E-02	3.73E-01
O14594	NCAN	0.183	0.088	3.93E-02	7.02E-01
O14793	GDF-8	0.095	0.047	4.42E-02	7.02E-01
P12544	GZMA	0.142	0.071	4.70E-02	7.02E-01
***APOE4* stratum**					
P55285	CDH6	0.638	0.171	3.33E-04	3.00E-02
Q16775	HAGH	0.479	0.147	1.59E-03	7.16E-02
Q92752	TN-R	0.280	0.094	3.72E-03	1.11E-01
P01138	Beta-NGF	0.431	0.182	2.00E-02	3.36E-01
Q8NFP4	MDGA1	0.164	0.070	2.22E-02	3.36E-01
P57087	JAM-B	0.318	0.137	2.24E-02	3.36E-01
P41217	CD200	0.335	0.155	3.31E-02	4.13E-01
Q9BS40	LXN	0.619	0.292	3.67E-02	4.13E-01
P16234	PDGF-R-alpha	0.324	0.159	4.42E-02	4.26E-01
***APOE33* stratum**					
Q2VWP7	PRTG	0.309	0.122	1.22E-02	7.70E-01
O94779	CNTN5	0.193	0.081	1.85E-02	7.70E-01
O14594	NCAN	0.248	0.110	2.57E-02	7.70E-01
***APOE2* stratum**					
P17405	SMPD1	-0.447	0.203	3.51E-02	9.66E-01
Q2TAL6	VWC2	-0.362	0.166	3.69E-02	9.66E-01

Abbreviations: β, regression coefficient; SE, standard error; *APOE*, apolipoprotein E; FDR, False discovery rate
*adjusted for age and sex

Supplementary Table 4: Results of interaction analysis of proteins with *APOE* in Rotterdam Study *(interaction P-value <0.05)*

Uniprot ID	annotation	$\beta_{interaction}$	SE	*P-value*$_{interaction}$
Q16775	HAGH	0.414	0.172	1.70E-02
P09919	G-CSF	0.276	0.125	2.78E-02
O95727	CRTAM	-0.221	0.106	3.77E-02

Abbreviations: β, regression coefficient; SE, standard error
Model: Alzheimer's disease ~ Protein levels * *APOE* ε4 (0/1) + Protein levels + *APOE* ε4 (0/1) + age + sex

Supplementary Table 5: Association of CSF levels of CDH6 levels with AD versus controls in the Amsterdam Dementia Cohort

**Stratum***	**Effect size (β)**	**SE**	***P-value***
**Overall sample**	0.329	0.220	***0.136***
***APOE4* stratum**	-0.107	0.476	*0.822*
APOE 33	0.457	0.363	*0.207*
APOE2 stratum	1.949	1.233	*0.114*

Abbreviations: AD, Alzheimer's disease; SE, standard error
*Logistic regression analysis adjusting for age and sex

Supplementary Table 6: Case-Control stratified association of CDH6 protein with CSF biomarkers of AD in the Amsterdam Dementia Cohort

Biomarker*	Effect size (β)	SE	***P-value***
**Controls only**			
Aβ-42	105.387	23.6	*<1x10^{-3}*
P-tau	10.03	1.22	*<1x10^{-3}*
t-tau	76.4	7.6	*<1x10^{-3}*
**AD only**			
*A*β-42	12.54	13.602	*0.36*
P-tau	27.757	4.496	*<1x10^{-3}*
t-tau	254.815	50.7	*<1x10^{-3}*

Abbreviations: AD, Alzheimer's disease; SE, standard error

*Linear regression analysis adjusting for age and sex

Supplementary Table 7: Results of association of protein levels with *APOE* genotypes

	APOE4 vs APOE3†			***APOE4 vs APOE2***‡			***APOE2 vs APOE3***α		
	**β**	**SE**	**P-values**	**β**	**SE**	**P-values**	**β**	**SE**	**P-values**
**Overall**									
**CDH6**	0.051	0.051	0.117	0.163	0.055	$3.79X10^{-3}$	-0.085	0.048	0.081
**HAGH**	-0.025	0.048	0.600	0.073	0.071	0.307	-0.091	0.077	0.237
**Cases only**									
**CDH6**	0.079	0.045	0.081	0.143	0.081	0.085	-0.005	0.081	0.945
**HAGH**	0.059	0.060	0.333	0.005	0.112	0.960	0.094	0.114	0.409
**Controls only**									
**CDH6**	-0.060	0.0505	0.235	0.061	0.072	0.406	-0.131	0.057	0.026
**HAGH**	-0.192	0.0860	0.028	0.002	0.081	0.972	-0.214	0.104	0.042

Abbreviations: β, regression coefficient; SE, standard error; *APOE*, apolipoprotein E

†*APOE* 44/34/24 *=1* versus *APOE* 33*=0*; ‡*APOE* 44/34=1 versus *APOE* 22/23=0; α*APOE* 22/23=1 versus *APOE* 33=0

Supplementary Table 8: Genome-wide significant variants ($P<2.5x10^{-8}$) from GWAS of CDH6 levels in plasma

rsID	Chr.	Position*	Alt.	Locus	Ref.	MAF	β	SE	P-values	R-square	Annotation	CADD
rs111283466	5	31130238	A	5p13.3	G	0.070	1.068	0.178	1.92E-09	0.985	Intergenic	0.292
rs10050733	5	31127060	C	5p13.3	T	0.070	1.061	0.177	2.13E-09	0.990	Intergenic	1.345
rs59671083	5	31126633	A	5p13.3	G	0.070	1.059	0.177	2.21E-09	0.991	Intergenic	8.567
rs13170907	5	31123473	C	5p13.3	T	0.070	1.050	0.176	2.54E-09	0.997	Intergenic	12.90
rs10071438	5	31114078	T	5p13.3	A	0.070	1.049	0.176	2.59E-09	0.997	Intergenic	7.310
rs10044808	5	31134528	T	5p13.3	C	0.061	1.085	0.188	7.97E-09	0.977	Intergenic	0.401
rs10044844	5	31134508	A	5p13.3	G	0.061	1.085	0.188	7.97E-09	0.977	Intergenic	0.729
rs10079279	5	31123143	G	5p13.3	A	0.061	1.053	0.185	1.24E-08	1.000	Intergenic	0.156
rs16900696	5	31113414	G	5p13.3	C	0.061	1.053	0.185	1.25E-08	0.998	Intergenic	6.323
rs28658063	5	31117714	C	5p13.3	G	0.061	1.053	0.185	1.25E-08	0.998	Intergenic	4.499
rs28702563	5	31117764	A	5p13.3	G	0.061	1.053	0.185	1.25E-08	0.998	Intergenic	4.673
rs7732113	5	31114502	A	5p13.3	G	0.061	1.053	0.185	1.25E-08	0.998	Intergenic	1.877
rs13358949	5	31112867	G	5p13.3	A	0.061	1.052	0.185	1.36E-08	0.996	Intergenic	1.647

Abbreviations: Chr., chromosome; MAF, Minor allele frequency; CADD, combined annotation dependent depletion.

* Basepair information is provided as per GRCh37/hg19

Note: variants are sorted as per P-values, β is coded as per alternative allele

Supplementary Table 9: Population characteristics of the BioFINDER study

	AD cases	Controls
**N**	186	485
**Age (SD), years**	75.1 (7.2)	70.6 (5.6)
**Female (%)**	63.90%	46.10%
**Body Mass index**	24.2	25.6
**MMSE (Mean)**	21.4	27.6
**Aβ42 (pg/mL)**	317	533
**t-tau (pg/mL)**	620	401
**p-tau (pg/mL)**	117	69
***APOE* genotype**		
***APOE* 44/34**	124	223
***APOE* 33**	58	224
***APOE* 22/23**	4	38

Abbreviations: AD, Alzheimer's disease, SD, Standard deviation, *APOE*, apolipoprotein E gene

Supplementary Table 10: Population characteristic of Amsterdam dementia Cohort (ADC)

	**AD cases**	**Controls**
**N (Male/Female)**	236 (140/95)	199 (118/81)
**Age (Mean±SD)**	66 (8)	58 (8)***
**MMSE (Mean±SD)**	19.7 (5)	28.2 (1.6) ***
**Aβ42 (pg/mL)**	600.1 (117.3)	1120 (212.3)***
**t-tau (pg/mL)**	770.5 (415.8)	214 (85.5)***
**p-tau (pg/mL)**	91 (37.2)	38 (85.5)***
***APOE* genotyped (N)**	219	150
***APOE* 44/34**	128	34
***APOE* 33**	79	93
***APOE* 22/23**	12	25

Abbreviations: AD, Alzheimer's disease
Note: Data are reported as medians and interquartile range unless otherwise indicated. *** *P-value* < 0.0001

Supplementary Figure 1:

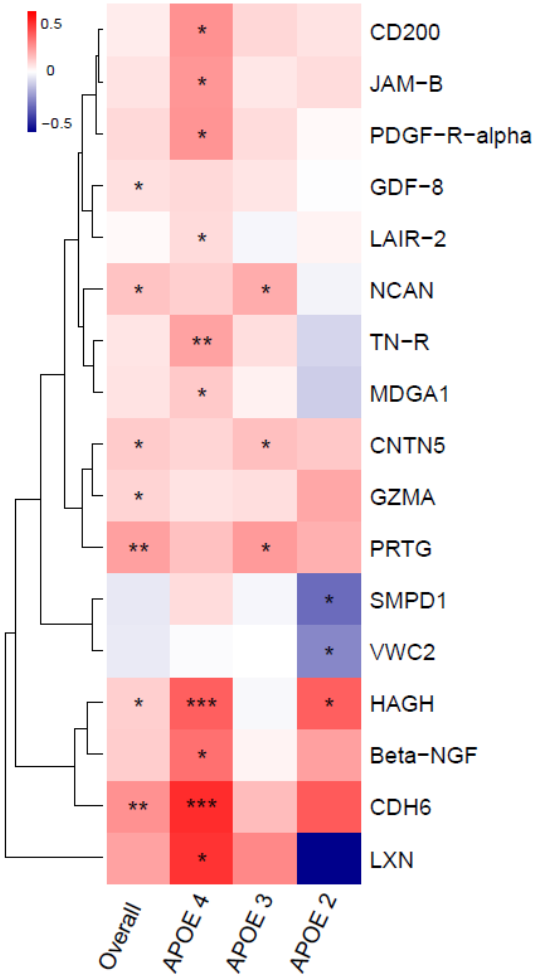

Supplementary Figure 2:

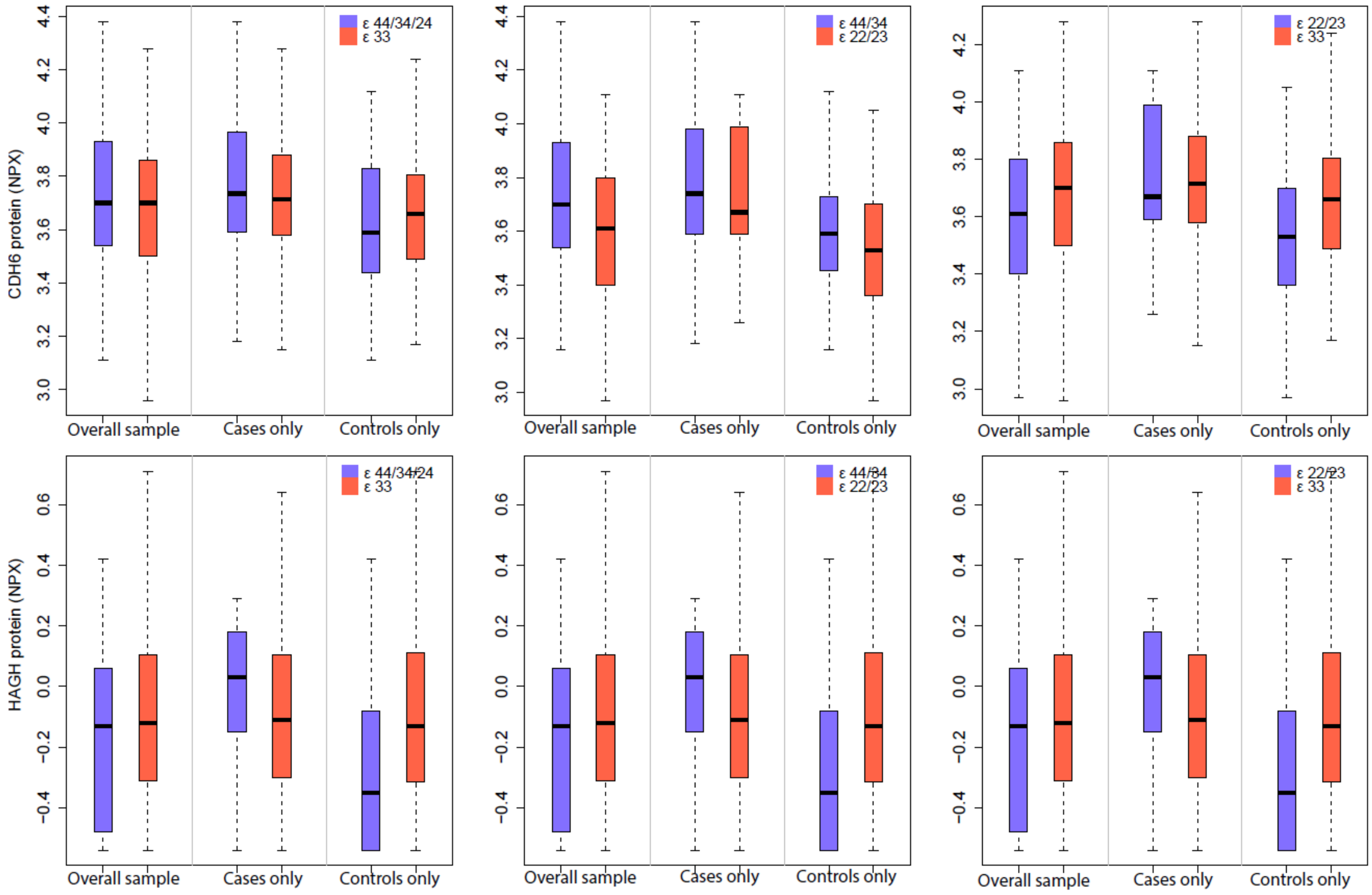

Supplementary Figure 3:

(a) **(b)**

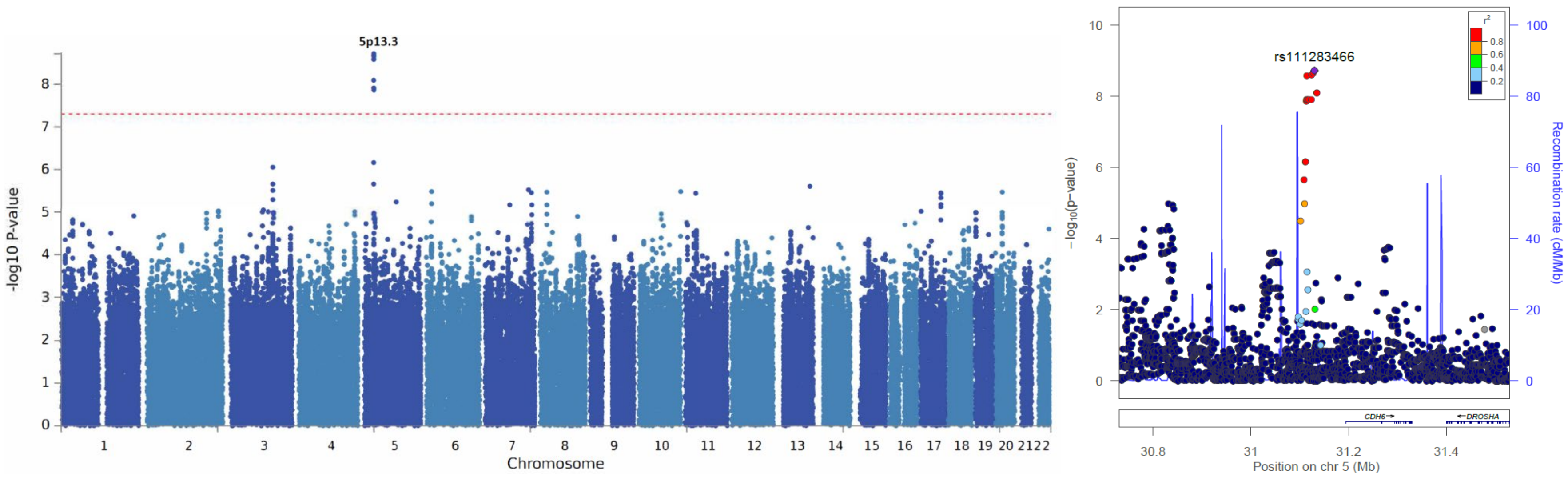

Supplementary Figure 4:

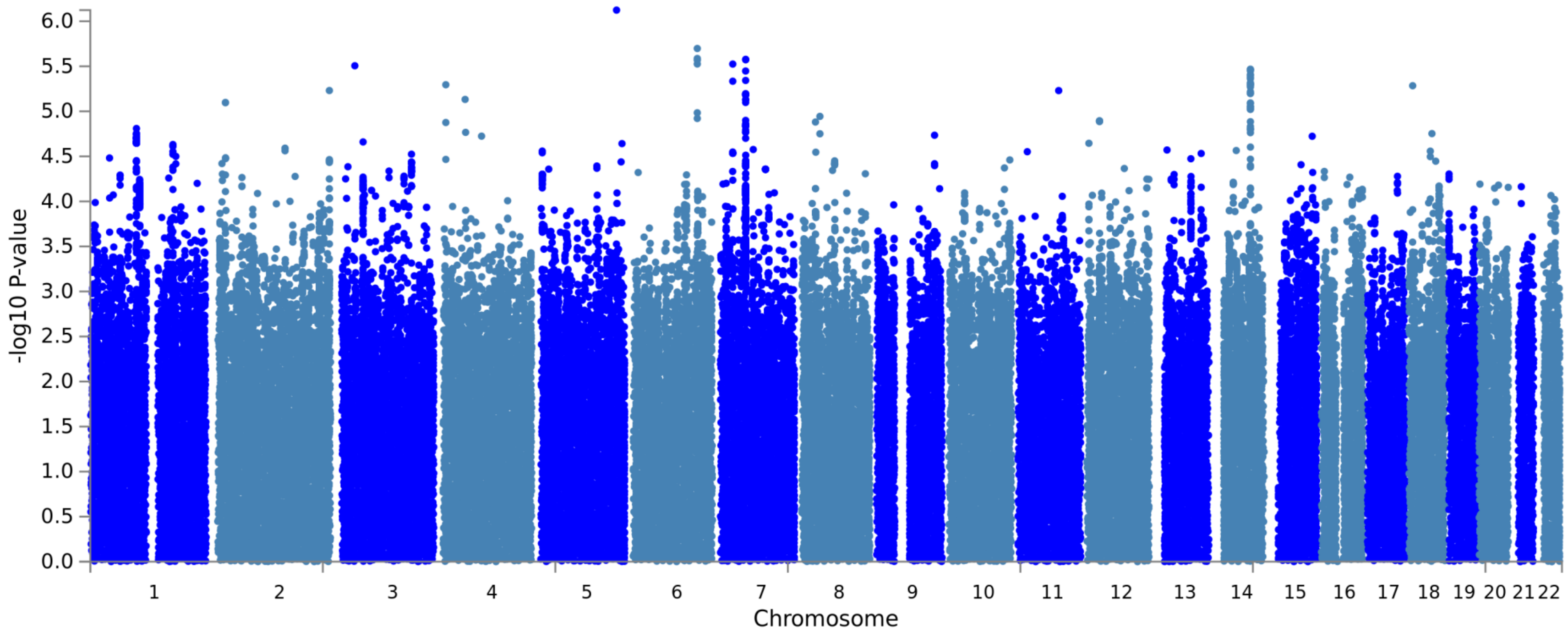

Supplementary Figure 5:

a)

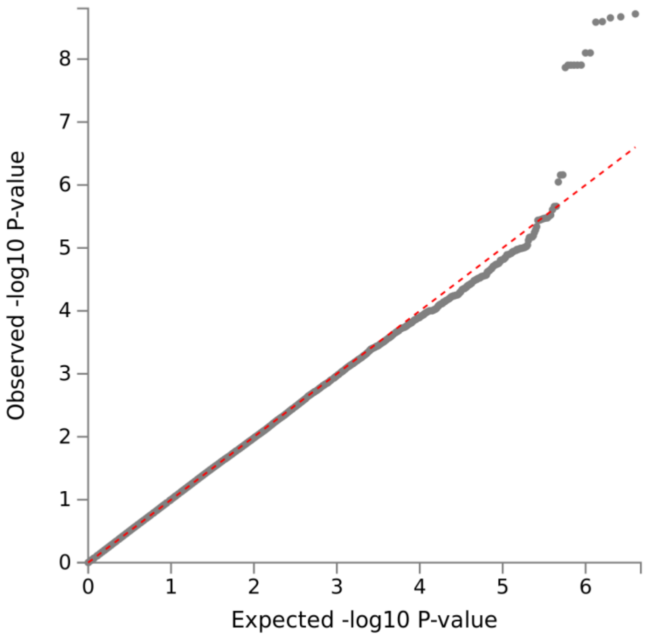

b)

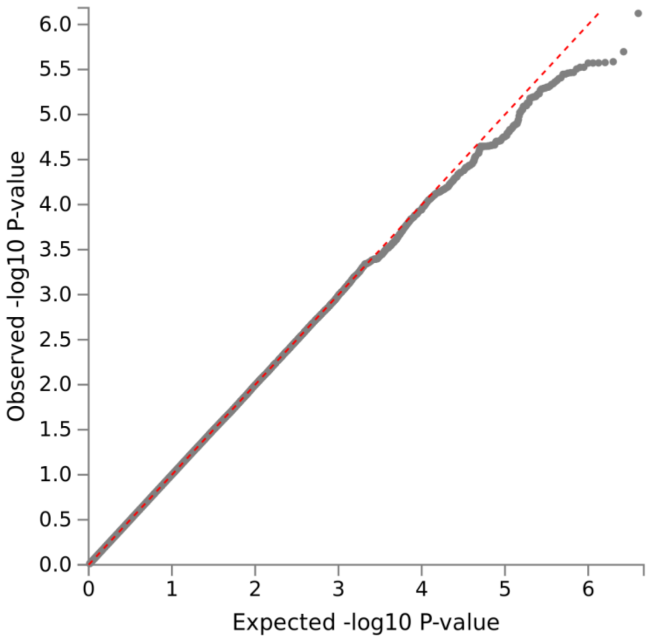

Supplementary Figure 1. Heatmap showing results of association protein levels with Alzheimer's disease (AD) reaching *P-value* < 0.05. Color of each box indicates the magnitude of effect size (β) of association and star notations represent the strength of association based on *P-values.*

Supplementary Figure 2. Boxplots representing the normalized protein expression (NPX) levels of CDH6 and HAGH among *APOE* 44/34/24 versus *APOE* 33; *APOE* 44/34 versus *APOE* 22/23; *APOE* 22/23 versus *APOE* 33 in overall study sample and stratified by case-control status.

Supplementary Figure 3. (a) Manhattan plot for the CDH6 protein levels in plasma. Observed association of all tested single nucleotide polymorphisms (SNPs) on autosomal chromosomes (*X-axis*) are displayed as –log10(P-values) on *Y-axis*. Red line indicates a genome-wide significant association ($P<2.5\times10^{-8}$) with CDH6 levels in plasma. Detected novel genome-wide significant loci 5p13.3 is led by rs11283466 SNP with *P value* = 1.92×10^{-9}. **(b)** Regional association plot for the association of rs11283466 with CDH6 protein levels in plasma. Plot represent all single nucleotide polymorphisms (SNPs) within 250kb region of lead SNP with height representing the –log10(P-values). Location of SNPs are based on NCBI build 37 and recombination rates are calculated based on the 1000 Genome project. Color scale represents the r2 values indicating linkage disequilibrium of SNPs in the region with lead SNP. Arrows on the genes denote the 5'-3' prime orientation.

Supplementary Figure 4. Manhattan plot for the genome-wide association study results of HAGH protein levels in plasma**.**

Supplementary Figure 5. Quantile-Quantile plots for genome-wide association study results of **(a)** CDH6; **(b)** HAGH protein levels in plasma.
